# Detection of *cfxA2*, *cfxA3*, and *cfxA6* genes in beta-lactamase producing oral anaerobes

**DOI:** 10.1590/1678-775720150469

**Published:** 2016

**Authors:** Buhle BINTA, Mrudula PATEL

**Affiliations:** University of the Witwatersrand, Faculty of Health Sciences, School of Oral Health Sciences, Department of Oral Biological Sciences, Division of Oral Microbiology, Gauteng, South Africa.

**Keywords:** Anaerobic bacteria, β-lactamases, Prevotella, Porphyromonas, β-lactams

## Abstract

**Purpose:**

The aim of this study was to identify β-lactamase-producing oral anaerobic bacteria and screen them for the presence of *cfxA* and BlaTEM genes that are responsible for β-lactamase production and resistance to β-lactam antibiotics.

**Material and Methods:**

Periodontal pocket debris samples were collected from 48 patients with chronic periodontitis and anaerobically cultured on blood agar plates with and without β-lactam antibiotics. Presumptive β-lactamase-producing isolates were evaluated for definite β-lactamase production using the nitrocefin slide method and identified using the API Rapid 32A system. Antimicrobial susceptibility was performed using disc diffusion and microbroth dilution tests as described by CLSI Methods. Isolates were screened for the presence of the β-lactamase-TEM (*BlaTEM*) and β-lactamase-*cfxA* genes using Polymerase Chain Reaction (PCR). Amplified PCR products were sequenced and the *cfxA* gene was characterized using Genbank databases.

**Results:**

Seventy five percent of patients carried two species of β-lactamase-producing anaerobic bacteria that comprised 9.4% of the total number of cultivable bacteria. Fifty one percent of β-lactamase-producing strains mainly *Prevotella*, *Porphyromonas*, and *Bacteroides* carried the *cfxA* gene, whereas none of them carried *blaTEM*. Further characterization of the *cfxA* gene showed that 76.7% of these strains carried the *cfxA2* gene, 14% carried *cfxA3*, and 9.3% carried *cfxA6*. The *cfxA6* gene was present in three *Prevotella* spp. and in one *Porphyromonas* spp. Strains containing *cfxA* genes (56%) were resistant to the β-lactam antibiotics.

**Conclusion:**

This study indicates that there is a high prevalence of the *cfxA* gene in β-lactamase-producing anaerobic oral bacteria, which may lead to drug resistance and treatment failure.

## INTRODUCTION

The human mouth harbours a complex microbial community containing aerobic and anaerobic bacteria. These bacteria cause polymicrobial opportunistic oral and extra oral infections. Anaerobic bacteria such as *Porphyromonas gingivalis*, *Treponema denticola*, *Fusobacterium nucleatum*, *Prevotella intermedia, Campylobacter rectus*, *Prevotella nigrescens*, *Parvimonas micra,* and *Eubacterium nodatum* cause periodontal diseases, odontogenic abscesses, orofacial infections, and have been implicated in brain abscesses^26^ ^,^ ^31^. Some of these bacteria are also isolated from sputum samples collected from patients with cystic fibrosis and ICU patients with aspiration pneumonia^17^ ^,^ ^21^. β-lactam antibiotics are often prescribed to treat these infections. However, studies have shown that many oral anaerobic bacteria have developed resistance to β-lactam antibiotics because of the production of β-lactamases^12^.

Bacterial resistance to β-lactam antibiotics has been attributed to resistance genes, present on chromosomal or plasmid DNA that can be transferred between commensal and pathogenic bacteria^30^. Studies have shown that there is a high prevalence of *cfxA* genes responsible for the β-lactamase production in *Prevotella* and *Capnocytophaga* species isolated from periodontal pockets^6^ ^,^ ^13^. In addition, c*fxA and cfxA2* genes have been isolated from oral infection sites as well as from the causative organisms isolated from these infection sites^7^ ^,^ ^16^. It has been suggested that this c*fxA/cfxA2* partition could be partly related to the genus and partly to the geographical origin of the enzyme-producing strains^9^. *CfxA2 and* c*fxA3* genes have been detected in anaerobic bacteria isolated from patients in France, United Kingdom, Norway, Argentina, and the United States of America^11^ ^,^ ^20^ ^,^ ^25^, whereas c*fxA6* have only been detected in anaerobic bacteria isolated from patients in Argentina^5^. No data are available from Africa on the production of β-lactamases by oral bacteria and the genes responsible for the production of these enzymes in patients with chronic periodontitis. This study was therefore conducted to isolate and identify β-lactamase-producing oral anaerobic bacteria from the periodontal pockets of patients with chronic periodontitis and to detect the genes responsible for the production of these enzymes.

## MATERIAL AND METHODS

### Study population

This study was conducted in the Oral Health Centre, Johannesburg in 2012. Forty-eight patients diagnosed with severe to moderate forms of chronic periodontitis using classification provided by the American Association of Periodontology^1^ and with pocket depths of five millimeters and more were invited to participate in the study. Ethics clearance was obtained from the Human Research Ethics Committee (certificate no.: M 110112) and written consent was obtained from all participants. Patients with a history of previous periodontal treatment, necrotizing ulcerative gingivitis, diabetes, or those who had taken systemic antimicrobials or anti-inflammatory drugs four weeks prior to the study were excluded.

After careful removal of supragingival plaque, a sterile paper point was inserted into the two deepest pockets and left in place for ten seconds. Paper points were pooled in 1 mL of reduced transport fluid and processed within an hour of sampling to ensure the viability of anaerobic bacteria.

### Isolation and identification of β-lactamase producing bacteria

The samples were vortexed for 30 s, serially diluted in phosphate buffered saline, and 0.1 mL of 1/10 to 1/10000 dilutions were plated on 5% blood agar plates supplemented with 5 mg/L of haemin (Sigma-Aldrich, Johannesburg, Gauteng, South Africa) and 1 mg/L of menadione (Sigma-Aldrich, Johannesburg, Gauteng, South Africa) for determining the total anaerobic bacterial count. The proportion of the microbiota resistant to amoxicillin was determined by plating samples on blood agar plates supplemented with 3 µg/mL of amoxicillin with and without 0.75 µg/mL of clavulanic acid^15^. The plates were anaerobically incubated for one week at 37^o^C and the number of colony forming units (cfu) was counted. The bacteria that grew on amoxicillin but not on the amoxicillin-clavulanic acid plate were identified using the API 32A system (BioMerieux, Midrand, Gauteng, South Africa) and evaluated for β-lactamase production using the Nitrocefin Paper Disc Spot test. Although other techniques can be used, the API 32A system was the only one available at the time.

### Detection of β-lactamase genes

DNA was extracted from β-lactamase producing isolates using a technique described by Handal, et al.^13^ (2005). A loopful of culture was inoculated into a sterile eppendorf tube containing 10 µL of 10× PCR buffer, 15 mM MgCl_2_ (Qiagen, Whitehead Scientific products, Johannesburg, South Africa), and 90 µL of sterile distilled water. The inoculated buffer was boiled at 95°C for 10 min, cooled on ice, and centrifuged at 5000 rpm for 10 min. The supernatant was harvested and stored at -70°C until required.

DNA was amplified using the Polymerase Chain Reaction (PCR) technique. A 25 µL reaction mixture containing 12.5 µL of 2× PCR Master Mix, ٢.٥ µL of sterile nuclease-free water, 5 µL of 5 µM primer (For bla_TEM_ GTATGGATCCTCAACATTTCCGTGTCG and ACCAAAGCTTAATCAGTGAGGCA, and for bla_CfxA_ GCAAGTGCAGTTTAAGATT and GCTTTAGTTTGCATTTTCATC) was added to 5 µL of template DNA. Samples were amplified in an iCycler thermal cycler (BIO-RAD, USA) using the PCR conditions described by Handal, et al.^12^ (2005) *Escherichia coli* ATCC 25746 (University of Copenhagen) was used as a positive control for amplification of *bla*_*TEM*_. The *P. intermedia* isolate containing the *bla*_*cfxA*_ gene was used as the positive control for the amplification of that gene. For both PCR reactions, the negative control consisted of sterile water. Amplified PCR products were isolated using gel electrophoresis, sequenced, and further characterized by the BLAST option of the nucleotide GenBank│European Molecular Biology Laboratory - databases.

### Antimicrobial susceptibility

All the 85 β-lactamase producing isolates were subjected to antimicrobial susceptibility using a disk diffusion test and the results were interpreted according to the Clinical and Laboratory Standards Institute (CLSI) performance standards for antimicrobial disk susceptibility tests^4^. Minimum inhibitory concentrations (MICs) were performed using the microbroth dilution method according to CLSI Methods for Antimicrobial Susceptibility Testing of Anaerobic Bacteria^3^. Only 17 strains were successfully revived and included in this assay.

The data were analyzed using the STATA statistical package (College Station, Texas, USA).

## RESULTS

### Demography and the prevalence of β-lactamase-producing anaerobic oral bacteria

**Figure 1:**
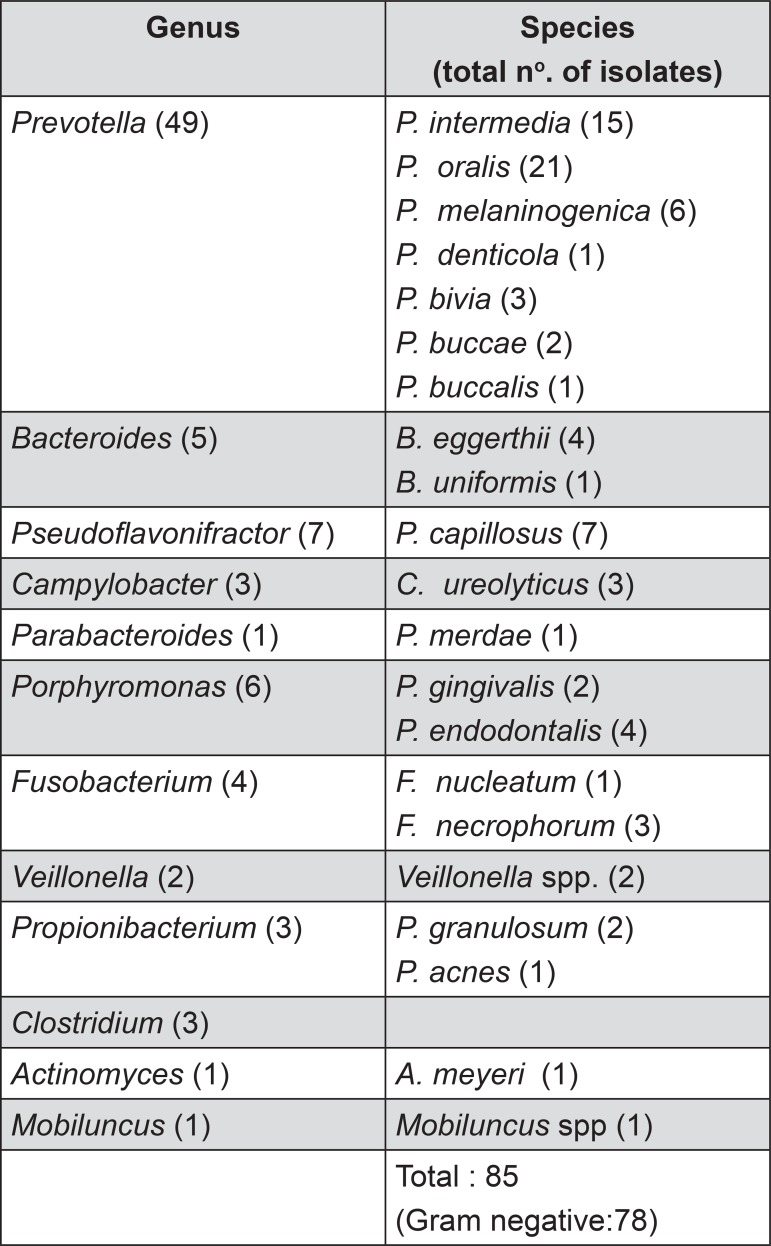
β-lactamase-producing oral anaerobic bacteria

The mean age of the patients was 52 with a range from 32 to 83 years of age. Fifty-eight percent of the patients were female. The average pocket depth of the sampled pockets was 7 mm with a range of 5 mm to 13 mm. Mean total anaerobic bacterial count on control plate was 1.8x10^6^ cfu/mL. Mean total anaerobic bacterial count on the amoxicillin plate was 1.9x10^5^ cfu/mL. Mean total anaerobic bacterial count on the augmentin plate was 5.9x10^4^ cfu/mL. Seventy-five percent of patients carried on average two species of β-lactamase-producing anaerobic oral bacteria, which constitute 9.4% of the total cultivable number of bacteria. All the isolates that grew on the blood agar plate containing amoxicillin tested positive for β-lactamase with the nitrocefin test. Seventy-eight of the 85 isolates of β-lactamase-producing bacteria were mainly gram negative black pigmented anaerobes (Figure 1).

### Detection of β-lactamase genes

Fifty-one percent of β-lactamase-producing isolates carried the *bla*_*CfxA*_ gene, whereas none carried *bla*_*TEM*_ (Table 1). Further characterization of the c*fxA* gene showed that 76.7% of these isolates carried the c*fxA2* gene (showing 100% similarity to *cfxA2* GenBank accession no. AM940016 of *Bacteroides ovatus)*, 14% carried the c*fxA3*gene (Genbank accession no. AY860640 of *Capnocytophaga ochracea* plasmid Pcap Mob A), and 9.3% carried c*fxA6* (Genbank accession no. FN376426 100% similarity with *Prevotella intermedia* partial *cfxA6* gene, strain PI51). Most of these isolates were from the black pigmented anaerobic bacteria such as *Prevotella* spp. and *Bacteroides* spp. The Minimal Inhibitory Concentration (MIC) for penicillin and amoxicillin was performed on the 17 isolates that carried c*fxA.* The results showed that up to 56% of these isolates were resistant to β-lactam antibiotics (Table 2).

**Table 1:** The prevalence of *cfxA* genes in the β-lactamase-producing oral anaerobic bacteria

β-lactamase gene (n=85)	Genus	Total n^o^. of	N^o^. of isolates with *cfxA* genes (%)		
		positive isolates (%)	*cfxA2*	*cfxA3*	*cfxA6*
*cfxA*	*Prevotella* spp	21	14	4	3
	*Porphyromonas* spp	4	3	-	1
	*Bacteroides* spp	12	10	2	-
	*Fusobacterium* spp	2	2	-	-
	*Clostridium* spp	2	2	-	-
	*Propionobacterium* spp	2	2	-	-
Total		43 (50.6)	33 (76.7)	6 (14)	4 (9.3)
*BlaTEM*	As above	0	-	-	-

**Table 2:** Antimicrobial susceptibility (Disc diffusion test) of β-lactamase-producing anaerobic bacteria against β-lactam antibiotics

	N^o^ of isolates resistant to β-lactam antibiotics (%)			
	^a^Disc diffusion test (n=85)			
*cfxA* gene	Ampicillin		Penicillin	
	**R**	**S**	**R**	**S**
Present (n=43)	18 (41.86)	25 (58.14)	24 (55.81)	19 (44.19)
Absent (n=42)	14 (33.33)	28 (66.67)	8 (19.05)	34 (80.95)

^a^Disc diffusion test (disk content 10 µg): R: Resistant ≤28 mm), S: Susceptible ≥29 mm)

### Antimicrobial susceptibility

All 85 β-lactamase-producing isolates were subjected to the disc diffusion test for ampicillin and penicillin. The results showed that 42 to 56% of the isolates containing resistance genes were resistant to β-lactam antibiotics (Table 2). MIC test, which is more accurate, showed that 53 to 59% of isolates that carried the resistance gene were resistant and another 6% had intermediate susceptibility to β-lactam antibiotics (Table 3).

**Table 3:** Antimicrobial susceptibility (Broth dilution test) of β-lactamase-producing anaerobic bacteria against β-lactam antibiotics

*cfxA* gene	N^o^ of isolates resistant to β-lactam antibiotics (%)					
	^a^Broth dilution test (n=17)					
	Amoxicillin			Penicillin		
	**R**	**I**	**S**	**R**	**I**	**S**
Present	9 (52.94)	0	8 (47.06)	10 (58.82)	1 (5.89)	6 (35.29)

^a^Broth dilution test: R: Resistant (≥2 µg/ml), I: Intermediate susceptibility (1 µg/ml), S: Susceptible (≤0.5 µg/ml)

## DISCUSSION

In this study, *Prevotella* spp. and *Bacteroides* spp. were the most prevalent β-lactamase-producing bacteria and 43% and 75% of these bacteria, respectively, carried the c*fxA* gene. These findings are similar to the results obtained in the North American, French, and Norwegian population^9^ ^,^ ^13^. The c*fxA* genes are known to be present in *Pseudomonas aeruginosa*, gut flora including bacteroides and oral bacteria such as *Prevotella* and *Capnocytophaga* ^2^ ^,^ ^8^ ^,^ ^13^. These genes are responsible for the resistance to penicillins and cephalosporins. The presence of these genes in oral commensals is always a cause for concern because this resistance can spread to serious pathogens and cause resistance to extended-spectrum cephalosporins as well. In addition, these normal commensals do cause extraoral infections which require antibiotic treatment. For example, *Capnocytophaga* spp containing the c*fxA3*-β-lactamase-producing gene have been isolated from patients with leukemia and neutropenia^22^ ^,^ ^23^. Similarly, c*fxA* gene-containing resistant *Prevotella spp* have been isolated from patients with cystic fibrosis and intra-abdominal infections^28^ ^,^ ^29^. The presence of these resistance genes in the oral microbiota could result in the commensals serving as reservoirs of antibiotic resistance^30^.

This study also showed that 77% of β-lactamase producing strains carried c*fxA2* genes. In the *Prevotella* spp, there was a predominance of the c*fxA* gene in North America, c*fxA2* in France^20^ ^,^ ^25^, and both genes in the United Kingdom^16^. Horizontal gene transfer might explain the spread of closely related gene sequences among these periodontal species^6^. Although the c*fxA3* gene has been found mainly in *Capnocytophaga* spp.^11^ ^,^ ^23^, in our study it was closely associated with *Prevotella* and *Bacteroides* spp. Interestingly, although c*fxA6* was initially detected in *Prevotella* spp. (Genbank accession no. FN376426) and also found in the present study, no other studies have detected this gene. In addition, the presence of c*fxA6* in *Porphyromonas* spp. has not been previously reported.

In this study, 10% of the periodontal pocket microbiota in 75% of the patients produced β-lactamase enzymes; and there were on average two species *per* patient. Of these β-lactamase producers, only 50.6% carried c*fxA* genes. However, oral bacteria that live in a biofilm environment, which is highly stressful and competitive, are known to adapt to genetic transfer. Metagenomic and bioinformatic studies have confirmed that oral bacteria play a major role in horizontal gene transfer^18^ because it improves their chance of survival, increases virulence, and alters their metabolism and drug resistance. Both plasmid and chromosomal-borne transfer of antibiotic resistance has been shown in oral bacteria^10^. Transformation through eDNA present in the plaque^14^ and membrane vesicles^24^ has also been described. In addition, a highly mobile Tn916, like as transposon that facilitates conjugation, has been found in many oral bacteria such as *Streptococci* species, *F. nucleatum*, *Eubacterium* species, *Veillonella* species, and *Actinobacillus* species. Therefore, a potential reservoir may transfer resistance genes to other drug sensitive bacteria from these 10% of drug resistant bacteria.

Some β-lactamase-producing anaerobes which carried β-lactamase genes were sensitive to β-lactam antibiotics (Table 3) with high MIC values of 0.125 µg/mL. This phenomenon has also been reported by other studies^30^ ^,^ ^32^. This is possibly because β-lactamases are ubiquitous in bacteria, and when produced in small quantities they may contribute little to antibiotic resistance, but could play a physiological role in peptidoglycan metabolism^19^. Garcia, et al.^8^ (2008) has also shown that c*fxA* gene containing *Bacteroides spp.* can show varying quantities of β-lactamase enzyme activity. In addition, some β-lactamases are constitutive whereas others require induction, which suggests that the mere presence of these genes may not contribute to the β-lactam antibiotics as shown by more reliable broth dilution techniques (53% and 59% resistance to amoxicillin and penicillin, respectively)^27^. Nevertheless, in nonresponsive patients antibiotics other than β-lactam should be considered. Our results also showed that many β-lactamase-producing bacteria were resistant to β-lactam antibiotics, but did not carry the c*fxA* or *Bla*_*TEM*_ gene, which suggests that there may be other genes responsible for the production of this enzyme (Table 2). This study only studied c*fxA* or *Bla*_*TEM*_ because these genes responsible for the production of β-lactamases are known to be present in oral bacteria. Other genes such as CepA/CblA are only occasionally identified in oral bacteria^9^.

One of the limitations of our study was the identification scheme. APi is an old technique based on biochemical reactions and was the only technique available in our laboratory at the time of this study. The identification percentage similarities of 80 and above were accepted as the final identification.

In conclusion, seventy-five percent of patients carried β-lactamase-producing anaerobic bacteria. C*fxA2,* c*fxA3, and* c*fxA6* genes were detected in 51% of these organisms, which comprised 10% of the total cultivable oral microbiota in our patients with chronic periodontitis. The c*fxA6* gene was found in *Prevotella* and *Porphyromonas* spp, which has epidemiological implications. Fifty-six percent of the isolates that carried c*fxA* genes were resistant to β-lactam antibiotics, which suggest that in nonresponsive patient antibiotics other than β-lactam should be considered.
